# Effects of different negative pressure wound therapy modes on wound healing: a systematic review

**DOI:** 10.3389/fmed.2026.1853536

**Published:** 2026-07-02

**Authors:** Cuiyi Wang, Xing Liu, Yitao Zhou, Naqin Liu, Yeqin Yang

**Affiliations:** Department of Nursing, Zhejiang Chinese Medical University, Hangzhou, Zhejiang, China

**Keywords:** negative pressure wound therapy, negative pressure wound therapy mode, systematic review, wound healing, wound management

## Abstract

**Introduction:**

Negative pressure wound therapy (NPWT) has been widely used in wound care, yet the optimal application mode—continuous, intermittent, or dynamic—remains unclear. This systematic review aimed to compare the effects of different NPWT modes on wound-healing-related outcomes and to provide evidence for mode selection in clinical practice.

**Methods:**

Following PRISMA guidelines, a systematic search was conducted across 11 databases—PubMed, MEDLINE, EMBASE, ProQuest, CINAHL, Web of Science, CENTRAL, CNKI, CBM, VIP, and WANFANG—from 1997 to 2024.

**Results:**

Ten clinical studies and 15 animal studies were included. Clinical studies involved both acute and chronic wounds, whereas animal studies mainly used full-thickness skin wound models. Reported outcomes included clinical efficacy, wound microenvironment, and molecular-level changes. Current evidence suggests that continuous, intermittent, and dynamic NPWT may influence wound healing in different ways. Compared with continuous NPWT, the two non-continuous modes—intermittent NPWT and dynamic NPWT—appeared to offer potential advantages in selected outcomes, particularly with respect to wound healing speed, tissue perfusion, and granulation-related responses.

**Discussion:**

Due to substantial heterogeneity in wound types, parameter settings, outcome measures, and study designs, the available evidence remains insufficient to establish a definitive ranking among NPWT modes or to determine optimal treatment parameters. Future research should focus on large-scale, high-quality, multicenter studies with refined wound-type stratification and greater standardization of pressure parameters and outcome measures to clarify the mode-specific effects, indications, and parameter settings of NPWT.

## 1 Introduction

Wound management remains a significant global healthcare challenge. A retrospective analysis of U. S. Medicare claim data identified approximately 10.5 million beneficiaries affected by chronic wounds in 2019, accounting for 16.3% of the total Medicare population, representing an increase of 1.8% since 2014 (1). Published studies have indicated that implementing appropriate wound interventions and management strategies can significantly reduce healthcare costs while improving clinical outcomes and patient quality of life (2). For example, effective management of diabetic foot ulcers can save between $85,000 and $1.1 million annually and can significantly reduce amputation rates (2). Since the invention of negative pressure wound therapy (NPWT) by Morykwas in 1997, this technique has been widely utilized for the management of various wounds (3–5). NPWT is a treatment system that applies sub-atmospheric pressure to the wound surface. The system generally comprises a polyurethane foam or gauze, an impermeable polyurethane adhesive film, an exudate collection system, and a suction pump (6). The mechanical deformation induced by negative pressure is regarded as the primary mechanism of NPWT, which includes macroscopic deformation (overall tissue traction and superficial tissue compression) and microscopic deformation (interaction between the dressing and the wound surface) (7). Macroscopic deformation enhances perfusion while reducing localized edema, whereas microscopic deformation facilitates granulation tissue formation and capillary network proliferation (7, 8). Other mechanisms include exudate management and stabilization of the local microenvironment (9). Current evidence demonstrates that NPWT exhibits significant efficacy, accelerating wound healing rates while reducing the incidence of complications (10–12). Furthermore, compared with conventional dressings, NPWT offers economic advantages and contributes to reducing overall societal healthcare costs (13, 14).

NPWT has three negative pressure modes: (a) Continuous NPWT (C-NPWT), which delivers a constant negative pressure throughout the treatment. (b) Intermittent NPWT (I-NPWT), which alternates between a set sub-atmospheric and atmospheric pressure (0 mmHg), maintaining atmospheric pressure for several minutes. (c) Dynamic NPWT (D-NPWT), which cycles between two set negative pressures generally without returning to 0 mmHg (e.g., −125 mmHg and −50 mmHg) (5). Despite its clinical utilization for over two decades, there is still no clear consensus on the optimal selection of negative pressure modes for wound healing. Morykwas found that I-NPWT increased granulation tissue formation by approximately 40% compared with C-NPWT (4). On the other hand, I-NPWT may lead to increased patient discomfort due to drastic changes in pressure associated with returning to atmospheric pressure (5, 15). Additionally, another study showed that the proliferation of *Escherichia coli* is higher under I-NPWT than under C-NPWT, potentially elevating the risk of infection (16).

Currently, in clinical practice, the selection of NPWT modes is predominantly based on empirical judgment or the routine adoption of C-NPWT. Systematic investigations comparing the therapeutic efficacy of I-NPWT, D-NPWT, and C-NPWT remain limited. The objective of this study was to systematically compare the impact of these three NPWT modalities on wound healing outcomes. It is anticipated that the findings will provide scientific guidance for the clinical application of NPWT, thereby enhancing therapeutic efficiency and improving patient comfort.

## 2 Methods

This systematic review was performed according to the Preferred Reporting Items for Systematic Reviews and Meta-Analyses (PRISMA) guidelines (17).

### 2.1 Literature search

This study involved searching across 11 databases, including seven English databases (CENTRAL, PubMed, MEDLINE, EMBASE, WOS, ProQuest, and CINAHL) and four Chinese databases (CNKI, WANFANG Data, CBM, and the VIP Database). The search covered the period from January 1997 to November 2024. Articles published since 1997 were included, as this year marked the first description of NPWT in its contemporary form. The initial keywords included “negative pressure wound therapy,” “vacuum-assisted closure,” “modes,” “modalities,” “continuous,” “intermittent,” and “dynamic.” For each database, the corresponding Medical Subject Headings (MeSH) terms or Emtree terms were identified and used, along with combinations of terms using Boolean operators (e.g., “AND” and “OR”). A detailed list of the search terms can be found in Supplementary material. The study was registered in the PROSPERO database (CRD42024608880) and was performed according to PRISMA guidelines (18).

### 2.2 Inclusion and exclusion criteria

The inclusion criteria for articles were as follows: (a) studies explicitly employing NPWT for wound management, with the intervention comparing at least two of the following modes: C-NPWT, I-NPWT, and D-NPWT; (b) wound healing outcomes were the primary outcomes, including but not limited to wound healing rates, inflammatory markers, and transcutaneous oxygen pressure (TcPO_2_); (c) the subjects were patients with a confirmed diagnosis of a specific disease or wound type, or animal models that had been standardized to simulate wounds; (d) the research design encompasses clinical trials (including randomized controlled trials, non-randomized controlled trials, cohort studies, and case–control studies) or animal studies with an appropriate control group; and (e) studies published in English or Chinese.

The exclusion criteria for articles were as follows: (a) duplicate publications; (b) studies with incomplete data; (c) studies with incomplete or erroneous data; (d) studies for which the full text was unavailable.

### 2.3 Literature screening and data extraction

Following the deduplication of search results, potential articles were screened based on titles and abstracts. Two authors independently screened full texts. Risk-of-bias assessment and data extraction were also performed independently by the same two authors. Any disagreements were resolved by a third author. We also screened the reference lists of all included articles. According to the research questions, data were extracted and entered into a data collection form. Outcomes included wound healing outcomes (e.g., healing rate and granulation tissue formation), wound microenvironment characteristics, and molecular-level alterations. The observation period for all outcomes did not exceed 3 months. Wound type, intervention modality, and relevant study characteristics were also extracted.

### 2.4 Risk-of-bias assessment

Risk-of-bias assessment was performed using the Risk of Bias Assessment Tool for Non-randomized Studies (RoBANS 2) for non-randomized studies (19), the Cochrane’s Risk of Bias 2 (ROB-2) tool for randomized clinical studies (20), and the Systematic Review Centre for Laboratory Animal Experimentation (SYRCLE) tool for animal model studies (21). The risk-of-bias assessment and quality assessment figures were produced with the help of the interactive online web application, “robvis.”

### 2.5 Certainty of evidence assessment

The certainty of evidence for each key clinical outcome was assessed using the Grading of Recommendations Assessment, Development and Evaluation (GRADE) framework, which evaluates evidence quality across five domains: risk of bias, inconsistency, indirectness, imprecision, and publication bias (22).

### 2.6 Statistical analyses

As the included studies exhibit considerable heterogeneity in research design, study subjects, and outcome indicators, it is difficult to conduct a quantitative analysis; therefore, only a qualitative analysis was conducted.

## 3 Results

### 3.1 Search results

**Figure 1:**
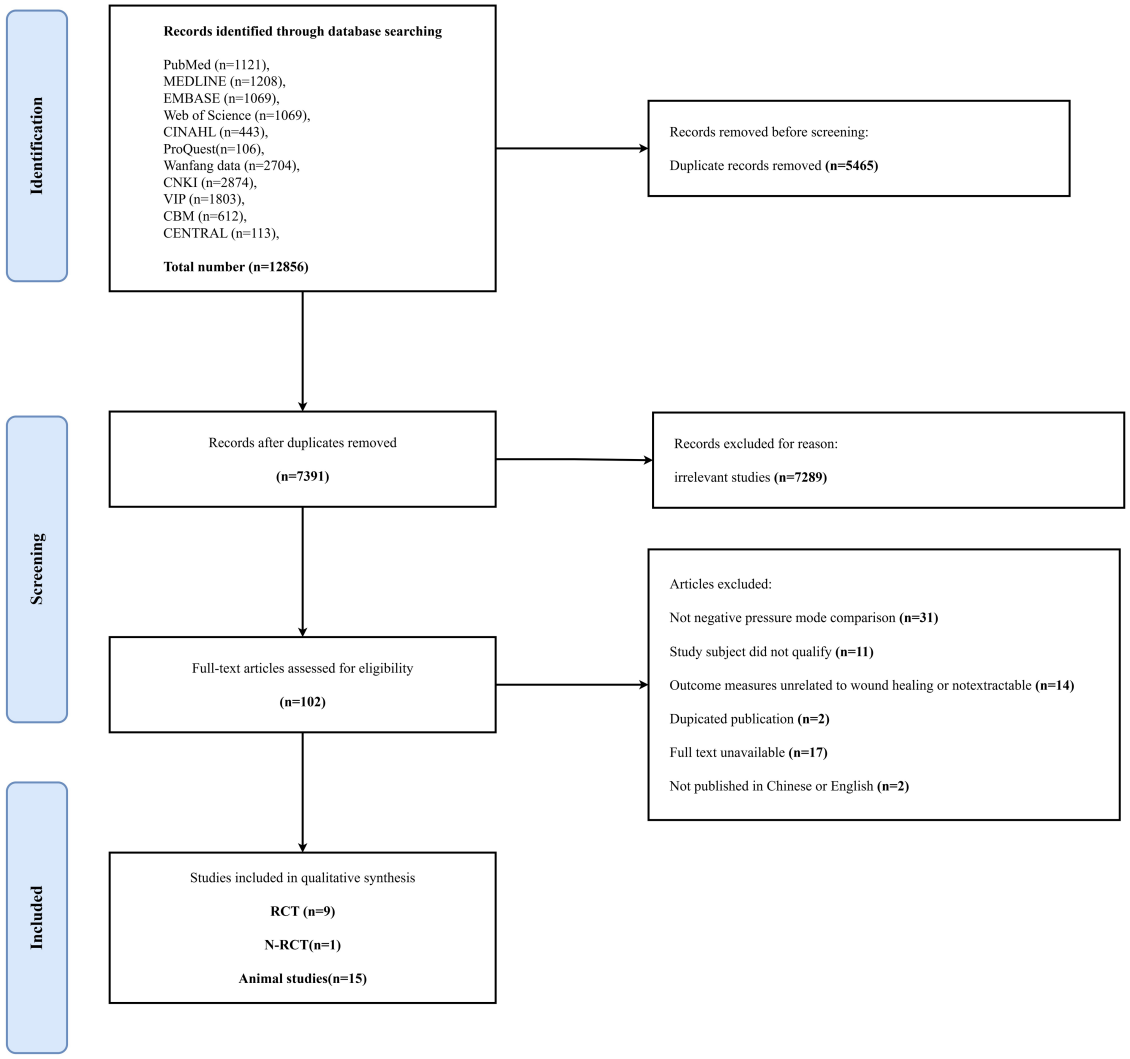
PRISMA flow diagram of the study selection process.

A total of 12,856 related records were initially retrieved. After removing duplicates, 7,391 articles remained. Subsequent title and abstract screening excluded 7,289 publications deemed irrelevant to the research objectives, resulting in 102 articles eligible for full-text assessment. Following a detailed evaluation, 25 studies ultimately met the inclusion criteria, including 10 clinical studies and 15 animal studies. Excluded studies (*n* = 77) were categorized according to the exclusion criteria: non-comparative studies of negative pressure modalities (*n* = 31), non-wound-related study designs (*n* = 11), outcomes unrelated to wound healing or with incomplete data reporting (*n* = 14), conference abstracts with unavailable full texts (*n* = 17), non-English/Chinese publications (*n* = 2), and duplicate publications (*n* = 2). A flow diagram of the study selection process is shown in Figure 1.

### 3.2 Study characteristics

Ten clinical studies [one non-randomized controlled trial (NRCT) (23) and nine randomized controlled trials (RCT) (15, 24–31)] conducted in China between 2009 and 2023 were included in this review, enrolling a total of 669 patients. These studies included various wound types: five studies on diabetic foot ulcers, one study on venous leg ulcers, one study on traumatic wounds, two studies on chronic wounds (involving wounds with healing times exceeding 4 weeks or non-healing wounds), and one study covering both acute and chronic wounds (wounds healing within 42 days were classified as acute and subacute, while those requiring over 42 days were categorized as chronic). Regarding interventions, nine studies compared C-NPWT with I-NPWT, while one compared D-NPWT with C-NPWT. A detailed summary of the basic characteristics of the included studies is provided in Supplementary material.

Fifteen animal studies (32–46) (10 porcine, four murine, and one rabbit model) published from 2010 to 2024 were analyzed. Five studies were carried out in China, while three studies were conducted in the United States. Additionally, two studies were carried out in each of South Korea, Indonesia, and Sweden, and one study was conducted in Japan. The wound types covered included full-thickness wounds (nine studies), skin-graft wounds (two studies), burns (three studies), and surgical incisions (one study). Regarding interventions, six studies compared C-NPWT with I-NPWT, three studies compared C-NPWT with D-NPWT, one study compared I-NPWT with D-NPWT, and five studies evaluated all modes. A detailed summary of the basic characteristics of the included animal studies is provided in Supplementary material.

Substantial heterogeneity was observed in outcome measures across the included clinical studies, while certain outcomes from animal studies lacked standardized quantification, precluding meta-analytic integration. Outcomes were categorized into three domains: clinical efficacy, wound microenvironment, and molecular-level changes. Clinical efficacy included healing outcomes, flap survival rate, healing speed, and pain assessment. The wound microenvironment encompasses wound bed formation quality, blood perfusion, wound bacterial load, and exudate evaluation. Nineteen studies reported clinical efficacy, 18 studies reported wound microenvironment outcomes, and 11 studies reported molecular-level changes. Clinical efficacy and wound microenvironment outcomes are presented in Table 1, while molecular-level changes are summarized in Table 2. To contextualize the strength of the available clinical evidence, the certainty of evidence for each key clinical outcome was assessed using the GRADE framework; as GRADE is designed for human clinical research, animal studies were not included in this assessment (22). A summary of findings is presented in Tables 3, 4, with detailed evidence profiles provided as Supplementary material.

**Table 1:** Clinical Efficacy and Wound Microenvironment.

Study		Clinical efficacy				Wound microenvironment					
		Healing effect	Flap survival rate	Healing speed	Pain	Wound bed formation quality			Tissue oxygenation and perfusion	Wound bacterial condition	Exudate
						Granulation tissue	Cell proliferation	Vascularization			
Clinical studies	Kaixuan et al. (2009) (25)			C=I						C=I	
	Wang et al. (2016) (26)			C=I		C < I					C=I
	Guoping et al. (2017) (28)			C < I	C < I					C=I	C=I
	Jieming (2019) (24)			C < I							
	Minlie et al. (2020) (30)			I=D	C < D					C=D	C=D
	Shi et al. (2022) (15)			C=I	C > I						
	Hongping (2022) (27)	C=I		C < I							
	Yang et al. (2023) (23)			C < I					C=D		
	Ji et al. (2023) (29)	C=I		C < I		C < I			C < I	C < I	
	Xiyou et al. (2022) (23)	C=I		C < I		C < I			C < I		
Animal studies	Borgquist et al. (2010) (8)								I=D		
	Dastouri et al. (2011) (32)					C=D	C=D	C=D			
	Malmsjo et al. (2012) (35)			C < (I=D)		C < (I=D)					
	Woo et al. (2013) (39)			C=I=D				C < I < D	I < D		
	Lessing et al. (2013) (41)			C=I=D		C=I=D					
	Jian et al. (2014) (45)			C < I < D							
	Lan et al. (2015) (46)			C=I=D			C=I	C=I			
	Xiaoming et al. (2017) (43)			C < I							
	Hong et al. (2019) (34)		C < D					C < D	C < D		
	Bin et al. (2020) (44)	C=I									
	Resadita et al. (2022) (37)			C=I							
	Minghan (2022) (42)					C < D	C < D	C < D			
	Seswandhan et al. (2023) (36)							C=I		C=I	
	Tsuchiya et al. (2024) (40)		C > I					C > I			

A comparison of the optimal results under the three modes is presented. The completed results are available in the Supplementary material.

**Table 2:** Molecular-level change.

Biomarkers		Studies suggesting no difference	Studies suggesting more changes in a specific mode		
			C-NPWT	I-NPWT	D-NPWT
Inflammatory markers	TGF-β1	Shi et al. (15), Yang et al. (30)			
	IL-1β	Shi et al. (15), Hongping (27), Yang et al. (30)			
	IL-6	Minlie et al. (30)		Shi et al. (15), Hongping (27)	
	IL-17			Hongping (27)	
	CRP			Hongping (27)	
Angiogenic markers	VEGF	Shi et al. (15), Minlie et al. (30), Tsuchiya et al. (40)		Xiaomin et al. (31), Seswandhana et al. (36)	Minghan (42)
	bFGF	Shi et al. (15), Minlie et al. (30)			
	FGF-2	Tsuchiya et al. (40)			
	TGF-β1	Shi et al. (15), Minlie et al. (30)			
	ET-1			Yang et al. (31)	
	Fit-1				Minghan (42)
	Ang1				Minghan (42)
	Ang2				Minghan (42)
Extracellular matrix remodeling	MMP	Wang et al. (26)			
	TIMP	Wang et al. (26)			
Oxidative stress markers	SOD			Yang et al. (31)	
	NO			Yang et al. (31)	
	MDA			Yang et al. (31)	
Metabolic enzymes	SDH			Ji et al. (29)	
	LDH			Ji et al. (29)	

**Table 3:** Summary of findings: I-NPWT vs. C-NPWT.

Outcome	Studies (n)	Summary of Findings	Certainty
Wound healing speed/rate	9 (~629)	I-NPWT faster in 6/9 studies (*p* < 0.05); 3/9 no difference; wound type and pressure settings are key effect modifiers	Very low
Overall healing effect (complete/effective rate)	4 (~310)	No significant difference in final healing outcome (4/4 studies, *p* > 0.05); both modes facilitate wound healing	Low
Granulation tissue/wound-bed quality	3 (~238)	I-NPWT superior granulation tissue formation in all three DFU studies (*p* < 0.05); evidence specific to DFU	Low
Tissue oxygenation/perfusion (TcPO2, blood flow)	2 (~198)	After 7 days: I-NPWT significantly higher TcPO2 and dorsal foot blood flow vs. C-NPWT (*p* < 0.05 to *p* < 0.01); DFU only	Very low
Pain (VAS score)	2 (~90)	Contradictory results: one study favors C-NPWT; another favors I-NPWT; superior mode indeterminate	Very low
Wound bacterial condition	4 (~300)	4/5 studies no significant difference; 1 DFU study I-NPWT superior clearance at day 7 (*p* < 0.05); both modes reduce bacterial load	Low
Exudate (volume, pH, composite)	2 (~90)	No significant difference between modes in exudate volume, pH, or composite exudate score	Very low
Inflammatory and growth factor markers	Multiple (~350)	Both modes reduce cytokines and increase growth factors; I-NPWT may induce greater IL-6/IL-17 reduction and VEGF increase; overall molecular trend similar	Low

**Table 4:** Summary of findings: D-NPWT vs. C-NPWT.

Outcome	Studies (*n*)	Summary of Findings	Certainty
Wound healing speed/rate	1 (*n* = 36)	No significant difference in healing rate at days 7 or 14 (*P* > 0.05); both modes are superior to routine dressing	Very low
Tissue oxygenation (TcPO2)	1 (*n* = 36)	D-NPWT (CANPT) significantly higher TcPO2 vs. C-NPWT at days 7 and 14 (*p* < 0.01)	Very low
Pain (VAS score)	1 (*n* = 36)	D-NPWT significantly lower pain vs. C-NPWT at day 7 (*p* < 0.05)	Very low
Wound pH and exudate	1 (*n* = 36)	D-NPWT: significantly lower wound pH and exudate volume vs. C-NPWT at days 7–14 (*p* < 0.05 to *p* < 0.01)	Very low
Inflammatory and growth factors	1 (*n* = 36)	Both modes reduce pro-inflammatory markers and increase growth factors vs. baseline; no significant between-mode difference in magnitude.	Very low

### 3.3 Risk-of-bias analysis

**Figure 2:**
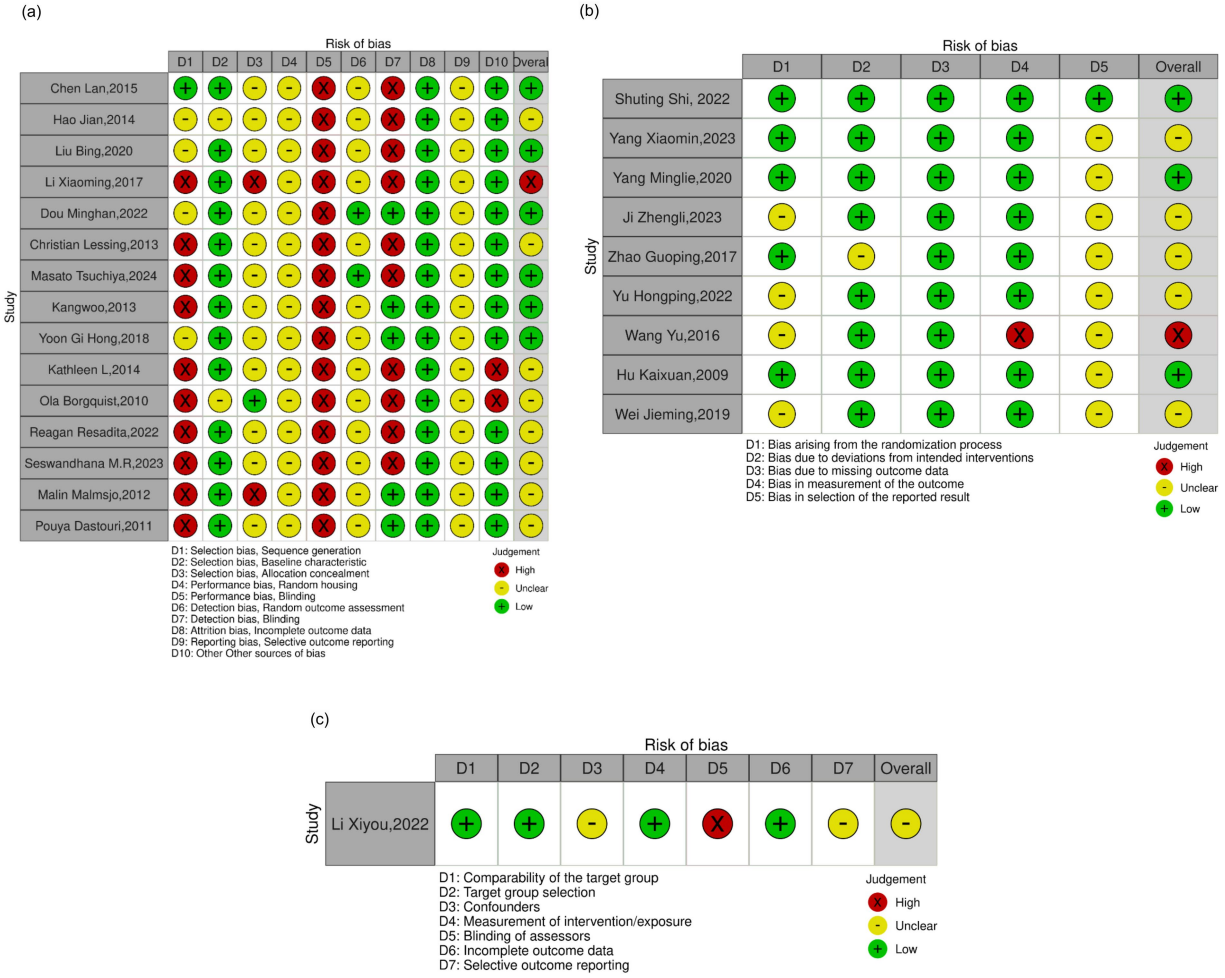
Risk-of-bias assessment of the included studies: **(a)** SYRCLE risk-of-bias assessment for animal studies; **(b)** Cochrane RoB 2 assessment for randomized controlled trials; **(c)** RoBANS 2 assessment for the non-randomized controlled trial. Green plus signs indicate low risk, yellow circles indicate unclear risk, and red crosses indicate high risk.

Animal studies were assessed using the SYRCLE tool, where six studies had a low risk of bias, eight studies were at moderate risk, and one study had a high risk of bias (Figure 2a). RCTs were assessed using the RoB 2 tool, with results indicating that three studies were at low risk, five studies were at moderate risk, and one study was at high risk (Figure 2b). NRCTs were evaluated using the RoBANS 2 tool, indicating that one study was at moderate risk (Figure 2c).

### 3.4 Clinical efficacy

#### 3.4.1 Healing effect

In clinical studies, the findings indicate that all NPWT modalities facilitate wound healing, with only one instance of treatment failure reported across the studies—this failure was attributed to severe limb ischemia, which is a contraindication for NPWT (25). Four studies (23, 27, 29, 31) categorized the final wound area or healing area into four grades: cured, markedly effective, effective, and ineffective, then calculated the healing effect rate. The results showed no significant difference between C-NPWT and I-NPWT (*p* > 0.05). An animal study (44) simulating surgical incisions reported no statistically significant difference (*p* > 0.05) in the number of cases achieving primary healing between the C-NPWT and I-NPWT groups. From the perspective of final wound-healing outcomes, all NPWT modes appeared to contribute to wound healing.

#### 3.4.2 Flap survival rate

Two animal studies have separately compared C-NPWT with I-NPWT and C-NPWT with D-NPWT, reporting flap survival rates. One study (40) found that, on day 9, the flap survival rate in the C-NPWT group was significantly higher than that in the I-NPWT group (*p* < 0.05). Another study (34) reported that, on day 10, the D-NPWT group exhibited a higher flap survival rate compared with the C-NPWT group (*p* < 0.05). Due to the lack of a direct comparison among all three modes, a definitive conclusion on flap survival rates in skin graft wounds cannot be drawn.

#### 3.4.3 Healing speed

Clinical studies measured wound healing rates (healing area/initial wound area), healing area, and ulcer area at different time points, and five studies also reported overall healing time. These indicators collectively reflect wound healing speed under different NPWT modes. In studies comparing C-NPWT and I-NPWT, six studies (23, 24, 27–29, 31) found that I-NPWT promoted faster healing (*p* < 0.05), while three studies (15, 25, 26) reported no significant difference (*p* > 0.05). Comparison between C-NPWT and D-NPWT showed no significant difference in healing speed (*p* > 0.05) (30).

Seven animal studies also evaluated healing speed. In pig models, four studies (37, 39, 41, 46) reported no significant difference in wound healing speed among different NPWT modes (*p* > 0.05). However, two studies reported positive findings: one found that, within 72 h, the I-NPWT and D-NPWT groups outperformed the C-NPWT group (*p* < 0.05) (35), while another study showed that, at 21 days, wound healing in the I-NPWT group was superior to that in the C-NPWT group (*p* < 0.05) (43). A study using a rabbit model simulating chronic ischemic wounds reported that, within 72 h, the D-NPWT group exhibited the fastest healing, followed by the I-NPWT group, with the C-NPWT group showing the slowest healing (*p* < 0.05) (45).

Existing studies suggest that wound healing speed differs among NPWT modes, with I-NPWT promoting faster healing than C-NPWT. However, clinical studies on D-NPWT are limited, and sample sizes remain insufficient to draw definitive conclusions. In animal studies, the relatively lower complexity compared with human wounds may account for the lack of significant differences in healing speed.

#### 3.4.4 Pain assessment

Pain outcomes varied across three clinical studies. Shi et al. (15) reported that on day 14, pain scores were significantly lower in the C-NPWT compared with the I-NPWT (*p* < 0.05). In contrast, Guoping et al. (28) found that pain scores were lower in the I-NPWT, but the observation time was not specified. The study comparing D-NPWT and C-NPWT demonstrated that on day 7, pain scores were lower in the D-NPWT group (*p* < 0.05) (30). However, two of the studies (15, 30) had small sample sizes, and the other (28) did not specify the observation time, highlighting the need for further validation and investigation.

### 3.5 Wound microenvironment

#### 3.5.1 Wound bed quality

The wound bed quality formation primarily encompasses three dimensions: granulation tissue formation, cell proliferation, and vascularization. Three clinical studies (23, 26, 29) focusing on diabetic foot ulcers reported granulation tissue conditions. The findings indicate that granulation tissue formation was superior in the I-NPWT compared with the C-NPWT (*p* < 0.05).

In animal studies, 10 studies reported this outcome, but results showed discrepancies, possibly due to differences in negative pressure settings and wound types. Among them, four studies compared three NPWT modes. Lee et al. (39) found that D-NPWT promoted the best vascularization, followed by I-NPWT, with C-NPWT being the least effective (*p* < 0.05). Hao et al. (45) and Malmsjö et al. (35) reported that I-NPWT and D-NPWT had similar effects on granulation tissue formation and cell proliferation, both outperforming C-NPWT (*p* < 0.05). However, Lessing et al. (41) found no significant differences among the three modes (*p* > 0.05). Additionally, three studies compared C-NPWT and I-NPWT. Tsuchiya et al. (40) found that C-NPWT improved vascularization more than I-NPWT (*p* < 0.05), while the other two studies (36, 46) showed no significant differences in cell proliferation and vascularization (*p* > 0.05). Furthermore, three studies examined the effects of C-NPWT and D-NPWT. Hong et al. (34) and Minghan et al. (42) found that D-NPWT performed better than C-NPWT in all three aspects (*p* < 0.05). Dastouri et al. (32) further examined different D-NPWT waveforms, showing that triangular-wave D-NPWT had similar effects to C-NPWT, both of which were better than square-wave D-NPWT (*p* < 0.05). They also found that longer intermittent durations improved wound bed formation.

In conclusion, existing studies do not determine which negative pressure mode is most effective for wound bed formation. However, different negative pressure modes, waveform selection, and wound types may all influence granulation tissue formation, vascularization, and cell proliferation.

#### 3.5.2 Tissue oxygenation and perfusion

In clinical studies, two studies (23, 29) on diabetic foot ulcers reported that after 7 days of treatment, the TcPO₂ in the I-NPWT group was significantly higher than in the C-NPWT group (*p* < 0.05). One study (29) further found that dorsal foot blood flow in the I-NPWT group was also significantly higher than in the C-NPWT group (*p* < 0.05). The study (30) comparing D-NPWT and C-NPWT showed no significant difference (*p* > 0.05), possibly due to limited sample size.

In animal studies, Borgquist et al. (38) measured blood flow changes in wounds and surrounding tissues under negative pressure. Their findings showed that when the low-pressure setting of D-NPWT was −10 mmHg, its effect on blood flow was similar to that of I-NPWT. Additionally, both modes exhibited a linear correlation between hemodynamic changes and pressure fluctuations. Woo et al. (39) found that D-NPWT (high negative pressure value: −125 mmHg, low negative pressure value: −50 mmHg) had the best perfusion among the three modes, while no significant difference was observed between C-NPWT and I-NPWT. Within the D-NPWT group, the −125/−50 mmHg setting outperformed other settings. Another study (34) on skin graft wounds showed that tissue perfusion in the D-NPWT group was significantly higher than in the C-NPWT group (*p* < 0.05).

Overall, NPWT effectively improves tissue perfusion, and I-NPWT and D-NPWT may promote periodic blood flow changes due to pressure fluctuations, enhancing wound oxygenation. This effect is particularly beneficial for ischemic wounds, such as diabetic foot ulcers. However, the optimal negative pressure parameters require further investigation.

#### 3.5.3 Wound bacterial condition

Five studies reported these wound bacterial conditions, including four clinical studies and one animal study. Only one study (29) indicated that after 7 days of NPWT treatment, bacterial clearance in the I-NPWT group was significantly greater than that in the C-NPWT group (*p* < 0.05). However, the remaining studies (25, 28, 30, 36) found no significant differences in bacterial reduction across different negative pressure modalities, with all treatment approaches effectively reducing bacterial load.

#### 3.5.4 Exudate

Three clinical studies compared wound exudate, with evaluation metrics including exudate volume, pH levels, and a composite score (assessing volume, color, viscosity, and odor). Two studies (26, 28) demonstrated no significant differences between C-NPWT and I-NPWT (*p* > 0.05), while another study (30) found no significant differences between D-NPWT and C-NPWT (*p* > 0.05).

### 3.6 Molecular-level changes

A total of 10 studies reported molecular-level changes. Overall, the three negative pressure modes showed a consistent trend in molecular regulation at the wound site, with differences observed only in the extent of specific molecular changes (see Table 2). This finding aligns with the gene and protein analysis conducted by Kathleen et al. in a pig model, which examined 84 genes and found that C-NPWT, D-NPWT, and I-NPWT regulated 56, 54, and 47 genes, respectively; furthermore, no significant differences were observed in key proteins, including Col1, VEGF, FN, VN, TNC, and CyCox (33).

Current evidence suggests that compared with C-NPWT, D-NPWT and I-NPWT induce greater changes in certain pro-inflammatory factors (e.g., IL-6, IL-17) and angiogenic factors (e.g., VEGF), while most molecular changes remain similar. However, these findings should be interpreted with caution due to limited sample sizes, inconsistent sampling times, and differences in negative pressure settings across studies.

## 4 Discussion

This systematic review compared the effects of three NPWT modalities on clinical efficacy, wound microenvironment, and molecular-level changes. Overall, the available evidence suggests that different NPWT modes may not be therapeutically equivalent. Compared with continuous negative pressure, non-continuous pressure delivery patterns, including intermittent and dynamic modes, appeared to show potential advantages in selected outcomes, particularly wound healing speed, tissue perfusion, and granulation-related responses. However, these findings were not consistent across all outcomes or studies, and the current evidence does not support a stable ranking of superiority among the three modalities. The GRADE assessment of the clinical evidence corroborated this conclusion, rating the certainty of evidence as very low to low across all key outcomes, driven in part by the inherent inability to blind participants and care providers in open-label NPWT modality comparisons.

The apparent advantages of non-continuous NPWT modes in selected outcomes may be related to differences in pressure delivery patterns. Compared with continuous NPWT, intermittent NPWT introduces periodic pressure fluctuations, which may contribute to greater changes in local perfusion and more pronounced stimulation of granulation-related responses, thereby favoring wound healing in some clinical settings (38, 47). Dynamic NPWT may share some of these features while maintaining sub-atmospheric pressure throughout treatment, which could partly explain its favorable performance in several animal studies, particularly in perfusion- and wound-bed-related outcomes (34, 41). In recent years, advances in mechanobiology have shown that cells are able to sense mechanical forces and respond through a range of biological processes, including morphological changes, migration, proliferation, and differentiation (48–50). This may also help explain why periodic pressure fluctuations during NPWT could induce different biological responses in wound tissues and thereby contribute to differences in therapeutic effects among treatment modes.

The findings of the included studies were not fully consistent, which may be partly explained by several factors. First, the available studies did not provide sufficiently refined comparisons across wound types, although different wounds may respond differently to NPWT modes. Some wounds may benefit more from improved perfusion and exudate control, whereas others may be more sensitive to mechanical stimulation and wound-bed remodeling. Second, variations in NPWT devices and wound management protocols may have contributed to differences in pressure parameters across studies, including pressure magnitude, low-pressure setting, cycle duration, and waveform pattern. In addition, the included studies selected different outcome measures and used different assessment methods, which made the findings difficult to compare directly and precluded quantitative synthesis. Although preclinical studies were included to complement the limited clinical evidence, the differences between animal wounds and human wounds should also be considered when interpreting the current evidence.

This review has several limitations. First, although an extensive search of both English and Chinese databases was conducted, all included clinical studies were from China. Only one conference abstract was identified from another region, and no subsequent study results were identified (51). In China, where medical resources are relatively constrained, NPWT is widely used, probably because it promotes wound healing while easing the burden on healthcare personnel (52–54). Consequently, healthcare professionals are more likely to adopt NPWT and focus on optimizing its application. Additionally, China’s relatively high population density may facilitate the implementation of such studies and the collection of clinical data. However, regional differences in healthcare systems, wound care protocols, NPWT devices, and patient characteristics may limit the generalizability of these findings. Second, many included studies had small sample sizes. A considerable proportion of the evidence was also assessed as having a moderate risk of bias. Finally, the restriction to Chinese- and English-language publications may have resulted in the omission of relevant studies published in other languages.

Future studies should include large-scale, multicenter, and rigorously designed clinical trials with more refined wound-type stratification. Given the complexity of waveform configurations and variability across NPWT devices, we recommend that future studies prioritize establishing the clinical advantage of intermittent negative pressure modes with clearly defined cycling parameters (e.g., 5 min of suction followed by 2 min of release) before investigating more complex waveform protocols. Greater standardization of pressure parameters, waveform definitions, and core outcome measures would help improve comparability across studies and facilitate quantitative evidence synthesis, thereby providing a stronger basis for mode-specific NPWT recommendations.

## 5 Conclusion

Current evidence suggests that different NPWT modes may influence wound healing outcomes in different ways. Compared with continuous NPWT, non-continuous modes, including intermittent and dynamic NPWT, may offer potential benefits in selected outcomes, particularly wound healing speed, tissue perfusion, and granulation-related responses. However, given the limited sample sizes, heterogeneous outcome measures, and the inclusion of animal studies, these findings should be interpreted with caution, and the current evidence is insufficient to establish a definitive ranking among modes or to determine optimal pressure parameters. Future research should focus on large-scale, high-quality, multicenter studies stratified by patient characteristics and wound type to clarify the differences, indications, and optimal parameter settings of different NPWT modes.

## Data Availability

The original contributions presented in the study are included in the article/Supplementary material, further inquiries can be directed to the corresponding author.
