# Supplementary material for: Effects of different negative pressure wound therapy modes on wound healing: a systematic review
Source: Front Med (Lausanne). 2026 Jul 2;13:1853536. doi: 10.3389/fmed.2026.1853536 (PMC13373038; doi:10.3389/fmed.2026.1853536)
Supplement: Supplementary file 1 [file Supplementary_file_1.DOCX]

# **1. Search terms**

| **Subject** | **Term** |
| --- | --- |
| Negative Pressure Wound Therapy | Negative-pressure wound therapy [MeSH Terms] |
|  | Negative pressure wound therap* |
|  | Negative-pressure wound therap* |
|  | Topical negative pressure therap* |
|  | Topical-negative pressure therap* |
|  | Vacuum assisted closur* |
|  | Vacuum-assisted closur* |
|  | Negative-Pressure Dressin* |
|  | Negative Pressure Dressin* |
|  | VAC therap* |
|  | V.A.C therap* |
|  | Vacuum dressing |
|  | TNP therap* |
|  | T.N.P therap* |
|  | Wound suction |
|  | Vacuum sealing therap* |
|  | Subatmospheric pressure therap* |
|  | Foam suction dressing |
|  | Sealed surface wound suction |
| Mode | Continuou* |
|  | Constan* |
|  | Sustain* |
|  | Intermitten* |
|  | Periodi* |
|  | Cyclic |
|  | Circula* |
|  | Dynami* |
|  | Variable |
|  | Noncontinuous |
|  | Traditiona* |
|  | mode |
|  | Modes |
|  | Redon drains |
|  | Wall suction |
|  | Single-use |

# **2. Search strategy**

**2.1 PubMed**

#1：

 Negative-pressure wound therapy[MeSH Terms]

#2：

 ((((((((((((((((('Negative pressure wound therap*'[Title/Abstract]) OR ('Negative-pressure wound therap*'[Title/Abstract])) OR ('Topical negative pressure therap*'[Title/Abstract])) OR ('Topical-negative pressure therap*'[Title/Abstract])) OR ('Vacuum assisted closur*'[Title/Abstract])) OR ('Vacuum-assisted closur*'[Title/Abstract])) OR ('Negative-Pressure Dressin*'[Title/Abstract])) OR ('Negative Pressure Dressin*'[Title/Abstract])) OR ('VAC therap*'[Title/Abstract])) OR ('V.A.C therap*'[Title/Abstract])) OR ('Vacuum dressing'[Title/Abstract])) OR ('TNP therap*'[Title/Abstract])) OR ('T.N.P therap*'[Title/Abstract])) OR ('Wound suction'[Title/Abstract])) OR ('Vacuum sealing therap*'[Title/Abstract])) OR ('Subatmospheric pressure therap*'[Title/Abstract])) OR ('Foam suction dressing'[Title/Abstract])) OR ('Sealed surface wound suction'[Title/Abstract])

#3：#1+#2

#4

(((((((((((((('Continuou*'[Title/Abstract]) OR ('Constan'[Title/Abstract])) OR ('Sustain*'[Title/Abstract])) OR ('Intermitten'[Title/Abstract])) OR ('Periodi*'[Title/Abstract])) OR ('Cyclic'[Title/Abstract])) OR ('Circula*'[Title/Abstract])) OR ('Dynami*'[Title/Abstract])) OR ('Variable'[Title/Abstract])) OR ('Noncontinuous'[Title/Abstract])) OR ('Traditiona*'[Title/Abstract])) ) OR ('mode'[Title/Abstract])) OR ('Modes'[Title/Abstract]))

#5: #3+#4

 ((Negative-pressure wound therapy[MeSH Terms]) OR (((((((((((((((((('Negative pressure wound therap*'[Title/Abstract]) OR ('Negative-pressure wound therap*'[Title/Abstract])) OR ('Topical negative pressure therap*'[Title/Abstract])) OR ('Topical-negative pressure therap*'[Title/Abstract])) OR ('Vacuum assisted closur*'[Title/Abstract])) OR ('Vacuum-assisted closur*'[Title/Abstract])) OR ('Negative-Pressure Dressin*'[Title/Abstract])) OR ('Negative Pressure Dressin*'[Title/Abstract])) OR ('VAC therap*'[Title/Abstract])) OR ('V.A.C therap*'[Title/Abstract])) OR ('Vacuum dressing'[Title/Abstract])) OR ('TNP therap*'[Title/Abstract])) OR ('T.N.P therap*'[Title/Abstract])) OR ('Wound suction'[Title/Abstract])) OR ('Vacuum sealing therap*'[Title/Abstract])) OR ('Subatmospheric pressure therap*'[Title/Abstract])) OR ('Foam suction dressing'[Title/Abstract])) OR ('Sealed surface wound suction'[Title/Abstract]))) AND ((((((((((((((('Continuou*'[Title/Abstract]) OR ('Constan'[Title/Abstract])) OR ('Sustain*'[Title/Abstract])) OR ('Intermitten'[Title/Abstract])) OR ('Periodi*'[Title/Abstract])) OR ('Cyclic'[Title/Abstract])) OR ('Circula*'[Title/Abstract])) OR ('Dynami*'[Title/Abstract])) OR ('Variable'[Title/Abstract])) OR ('Noncontinuous'[Title/Abstract])) OR ('Traditiona*'[Title/Abstract])) ) OR ('mode'[Title/Abstract])) OR ('Modes'[Title/Abstract]))

**2.2 WOS**

(TS=("Negative-pressure wound therap*" or "Negative pressure wound therap*" or "Topical negative pressure therap*" or "Topical-negative pressure therap*" or "Vacuum assisted closur*" or "Vacuum-assisted closur*" or "Negative-Pressure Dressin*" or "Negative Pressure Dressin*" or "VAC therap*" or "V.A.C therap*" or "Vacuum dressing" or "TNP therap*" or "T.N.P therap*" or "Wound suction" or "Vacuum sealing therap*" or "Subatmospheric pressure therap*" or "Foam suction dressing" or "Sealed surface wound suction" or "Negative-pressure wound therapy"))

AND

AB=("Continuou*" or "Constan*" or "Sustain*" or "Intermitten*" or "Periodi*" or "Cyclic" or "Circula*" or "Dynami*" or "Variable" or "Noncontinuous" or "Traditional" or "mode" or "Modes" or "Redon drains" or "Wall suction" or "single-use")

**2.3 CENTRAL**

#1 negative-pressure wound therapy（mesh）

#2 'Continuou*' or 'Constan*' or 'Sustain*' or 'Intermitten*' or 'Periodi*' or 'Cyclic' or 'Circula*' or 'Dynami*' or 'Variable' or 'Noncontinuous' or 'Traditional' or 'mode' or 'Modes' or 'Redon drains' or 'Wall suction' or 'single-use'

#3：#1+#2

**2.4 CINAHL**

AB: "Negative-pressure wound therap*" or "Negative pressure wound therap*" or "Topical negative pressure therap*" or "Topical-negative pressure therap*" or "Vacuum assisted closur*" or "Vacuum-assisted closur*" or "Negative-Pressure Dressin*" or "Negative Pressure Dressin*" or "VAC therap*" or "V.A.C therap*" or "Vacuum dressing" or "TNP therap*" or "T.N.P therap*" or "Wound suction" or "Vacuum sealing therap*" or "Subatmospheric pressure therap*" or "Foam suction dressing" or "Sealed surface wound suction" or "Negative-pressure wound therapy"

AND

AB: "Continuou*" or "Constan*" or "Sustain*" or "Intermitten*" or "Periodi*" or "Cyclic" or "Circula*" or "Dynami*" or "Variable" or "Noncontinuous" or "Traditional" or "mode" or "Modes" or "Redon drains" or "Wall suction" or "single-use"

**2.5 EMBASE**

Tita：'Negative-pressure wound therap*' or 'Negative pressure wound therap*' or 'Topical negative pressure therap*' or 'Topical-negative pressure therap*' or 'Vacuum assisted closur*' or 'Vacuum-assisted closur*' or 'Negative-Pressure Dressin*' or 'Negative Pressure Dressin*' or 'VAC therap*' or 'V.A.C therap*' or 'Vacuum dressing' or 'TNP therap*' or 'T.N.P therap*' or 'Wound suction' or 'Vacuum sealing therap*' or 'Subatmospheric pressure therap*' or 'Foam suction dressing' or 'Sealed surface wound suction' or 'Negative-pressure wound therapy'

And

Tita：'Continuou*' or 'Constan*' or 'Sustain*' or 'Intermitten*' or 'Periodi*' or 'Cyclic' or 'Circula*' or 'Dynami*' or 'Variable' or 'Noncontinuous' or 'Traditional' or 'mode' or 'Modes' or 'Redon drains' or 'Wall suction' or 'single-use'

**2.6 Chinese Database (CNKI, CBM, VIP, WANFANG Data)**

Search strategy of Chinese database is the same as above.
